# A comparative assessment of clinical whole exome and transcriptome profiling across sequencing centers: implications for precision cancer medicine

**DOI:** 10.18632/oncotarget.9184

**Published:** 2016-05-05

**Authors:** Eliezer M. Van Allen, Dan Robinson, Colm Morrissey, Colin Pritchard, Alma Imamovic, Scott Carter, Mara Rosenberg, Aaron McKenna, Yi-Mi Wu, Xuhong Cao, Arul Chinnaiyan, Levi Garraway, Peter S. Nelson

**Affiliations:** ^1^ Broad Institute of Massachusetts Institute of Technology and Harvard, Cambridge, 02142, MA; ^2^ Michigan Center for Translational Pathology, University of Michigan Medical School, Ann Arbor, 48109, MI; ^3^ Department of Urology, University of Washington, Seattle, 98195, WA; ^4^ Department of Medicine, University of Washington, Seattle, 98195, WA; ^5^ Department of Laboratory Medicine, University of Washington, Seattle, 98195, WA; ^6^ Divisions of Human Biology and Clinical Research, Fred Hutchinson Cancer Research Center, Seattle, 98109, WA

**Keywords:** precision oncology, genomics, sequencing, prostate cancer

## Abstract

Advances in next generation sequencing technologies provide approaches to comprehensively determine genomic alterations within a tumor that occur as a cause or consequence of neoplastic growth. Though providers offering various cancer genomics assays have multiplied, the level of reproducibility in terms of the technical sensitivity and the conclusions resulting from the data analyses have not been assessed.

We sought to determine the reproducibility of ascertaining tumor genome aberrations using whole exome sequencing (WES) and RNAseq. Samples of the same metastatic tumors were independently processed and subjected to WES of tumor and constitutional DNA, and RNAseq of RNA, at two sequencing centers. Overall, the sequencing results were highly comparable. Concordant mutation calls ranged from 88% to 93% of all variants including 100% agreement across 154 cancer-associated genes. Regions of copy losses and gains were uniformly identified and called by each sequencing center and chromosomal plots showed nearly identical patterns. Transcript abundance levels also exhibited a high degree of concordance (r^2^ ≥ 0.78;Pearson). Biologically-relevant gene fusion events were concordantly called. Exome sequencing of germline DNA samples provided a minimum of 30X coverage depth across 56 genes where incidental findings are recommended to be reported. One possible pathogenic variant in the *APC* gene was identified by both sequencing centers.

The findings from this study demonstrate that results of somatic and germline sequencing are highly concordant across sequencing centers that have substantial experience in the technological requirements for preparing, sequencing and annotating DNA and RNA from human biospecimens.

## INTRODUCTION

Rapid advancements in next generation sequencing (NGS) technologies have provided a means to comprehensively determine the constitutional genome of an individual, and all genomic aberrations within a tumor that occur as a cause or consequence of malignant growth. This information, when integrated with an understanding of disease mechanism, disease behavior, and response to therapeutics underlies the concept of precision oncology: a refinement of disease taxonomy based on molecular features [1]. A practical consequence of this approach is the development of a more specific categorization of cancers with congruent behaviors and with predictable responses to therapies.

The Cancer Genome Atlas (TCGA) and other large-scale molecular profiling studies of human malignancies have identified common and rare genomic alterations, a subset of which are recurrent across different tumor types and several of which have clear therapeutic implications [2, 3]. It is now evident that carcinomas typically contain thousands of mutations and a spectrum of structural chromosomal rearrangements and epigenomic alterations, a subset of which alter gene function and influence malignant growth [4]. While only a few molecular alterations have clearly-defined implications for selecting specific therapies, these are none-the-less notable, and foreshadow the future where new treatments are developed and deployed based on targeting key causal aberrations [5–7].

The field of medical genetics is currently grappling with the opportunities and challenges of integrating genomic sequencing data into clinical practice [8, 9]. Most commonly, whole genome sequencing (WGS) or whole exome sequencing (WES) strategies have been employed to identify sequence variants shown in clinical research to cause or associate with a disease. Setting standards for methods, determining which disease-associated variants should be reported, and establishing how to communicate incidental or opportunistic findings have been the subject of several consensus panels [10, 11]. Less attention has been given to specifically establishing standards for assessing and reporting somatic events in patients with cancer, which provide many additional challenges [12, 13].

In addition to the issues faced in interpreting and reporting germline WGS/WES data, cancer genomics must consider tissue quality and quantity, intra-tumor heterogeneity, inter-tumor heterogeneity, gains and losses of chromosomal regions, and variation in the admixture of neoplastic versus benign cells in the tissue sample. The addition of complementary NGS assays such as RNA sequencing (RNAseq) for the analysis of gene expression adds additional variables. If comprehensive genome-scale assessments will be used as the basis for cancer classification and consequent treatment decisions, then accurate molecular assessments are essential.

Although providers offering cancer genomics assays have proliferated, the level of reproducibility in terms of the technical sensitivity and the resulting conclusions stemming from the data analysis has not been evaluated. In this study, we sought to determine the reproducibility of NGS-based assessments of tumor genome mutations and gene expression using WES and RNAseq, respectively. Samples of the same metastatic tumors were independently processed and subjected to WES and RNAseq at two sequencing centers. We compared the determinations of somatic DNA point mutations, indels, and copy number variants identified by WES, germline variants assessed by WES, and transcript abundance and gene rearrangements identified by RNAseq.

## RESULTS

### Clinical samples, pathology, and sequence analysis pipelines

**Figure 1:**
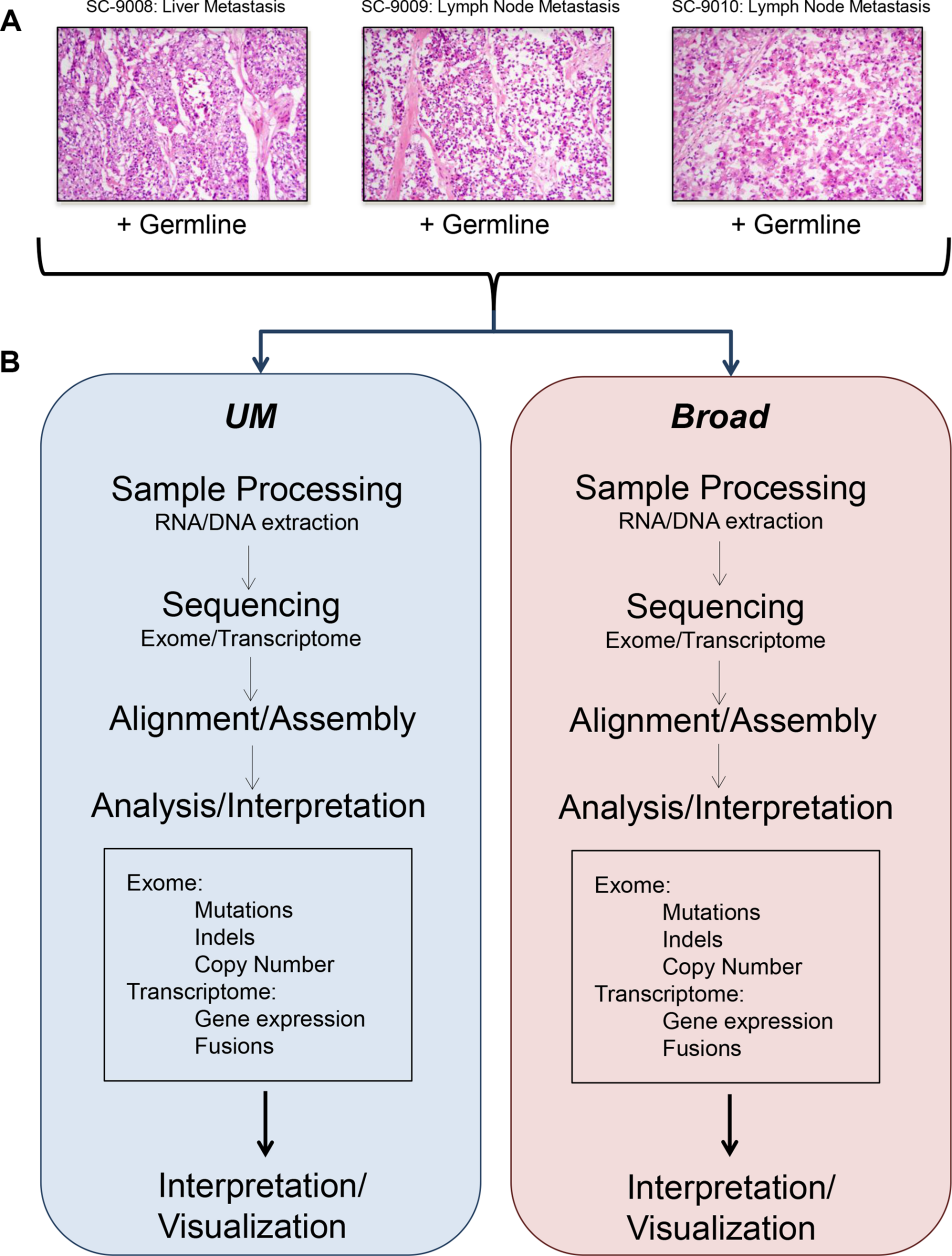
Flow of experiments and analyses Representative histology images from the tumor samples included in this study (**A**). Overview of sample processing, sequencing, and analysis pipelines used at the two sequencing centers (**B**).

Tumor and benign tissue samples were obtained from three men with widely metastatic prostate cancer [14, 15]. Tumor sections were assessed to confirm a composition of > 70% tumor cells, and benign tissues were evaluated to establish the absence of neoplastic cells. A metastasis from each patient, designated SC_9008 (liver), SC_9009 (lymph node) and SC_9010 (lymph node) was partitioned and one representative tumor sample from each metastasis with a corresponding benign tissue sample was shipped to the University of Michigan (UM) and the Broad Institute (Broad) for sequence analysis (Figure 1).

Each sequencing center processed the tissue samples using institutional protocols (see Methods). Exome libraries were sequenced using Illumina HiSeq instruments with a target of 50 million paired-end reads per sample. The actual number of reads ranged from 110 to 250 million (Table 1). RNAseq libraries were constructed from both PolyA+ selected and total RNA libraries (UM) or total RNA alone (Broad) and sequenced using Illumina HiSeq 2500 instruments with a target of 50 million paired-end reads per sample. The actual number of reads ranged from 100 to 134 million (Table 1).

**Table 1:** Sequencing metrics

Sequence Type	Metric	SC_9008		SC_9009		SC_9010	
		Broad	UM	Broad	UM	Broad	UM
**WES (somatic)**							
	MTC	177.99	264.10	147.09	271.13	122.57	265.04
	Selected bases (%)	0.83	0.75	0.84	0.76	0.85	0.72
	Zero coverage targets (%)	0.014	0.018	0.015	0.019	0.015	0.017
	NSV	852	1203	42	57	47	90
	Point Mutations	652	811	38	47	45	82
	Insertion/Deletions	200	392	4	10	2	8
**WES (germline)**							
	MTC	143.40	226.97	173.84	236.99	122.57	188.19
	Selected bases (%)	0.85	0.79	0.86	0.81	0.85	0.75
	Zero coverage targets (%)	0.014	0.020	0.013	0.021	0.015	0.020
	ACMG 56 gene coverage > 30X (%)	100	100	100	100	100	100
**RNAseq (somatic)**							
	Aligned in pairs reads (%)	0.976	0.912	0.973	0.914	0.974	0.920
	PF reads aligned (%)	0.938	0.821	0.948	0.819	0.940	0.812

MTC, mean target coverage; NSV, non-synonymous nucleotide variant.

### Sequencing coverage

**Figure 2:**
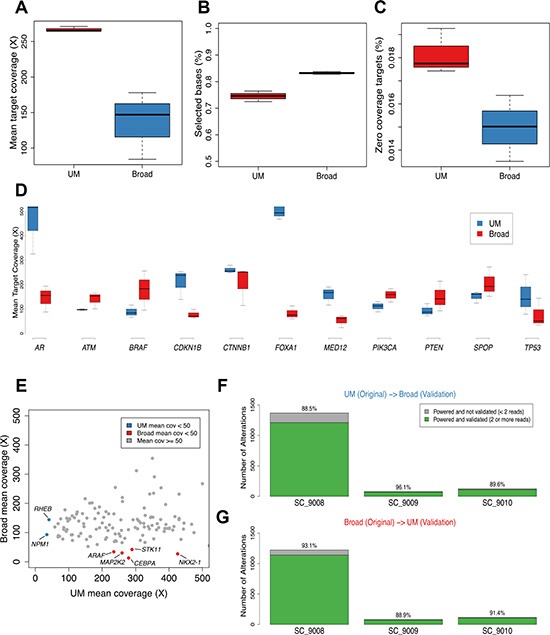
Sequence coverage of comparisons of mutation calls in prostate cancer across sequencing centers The range of mean target coverage (**A**), selected bases (%) (**B**), and zero coverage targets (%) (**C**) for tumors sequenced at the two sequencing centers are shown. Mean target coverage for biologically relevant prostate cancer genes are from tumors sequenced in the two sites are shown (**D**). Using a larger panel of 130 clinically relevant genes, mean target coverage for UM and Broad tumors is plotted in (**E**), with designations for genes that had < 50 X mean target coverage for UM (blue) or Broad (red) platforms. The cross validation rates for UM to Broad and Broad to UM are shown in (**F**) and (**G**), respectively when accounting for whether there was adequate power to detect an alteration at both sites which corrects for the difference in sequencing depth achieved between the two centers.

A mixture model was used to estimate the tumor cell content of each sample (eMethods). Histological assessments estimated that each tumor comprised > 70% neoplastic cells. The sequence-based estimates of tumor content were 86%, 76% and 58% for SC_9008, SC_9009 and SC_9010, respectively (Supplementary Figure 1). Mean target coverage and additional sequencing metrics are in Table 1 and Figure 2A–2C.

To compare the biological utility of exploring prostate cancer metastasis by WES, we assessed the mean sequencing depth of coverage for 11 genes shown in previous studies of prostate carcinoma to be recurrently mutated (Figure 2D) [16–18]. Each gene had a minimum of 50 reads spanning each nucleotide across the targeted exons. For the androgen receptor (AR) the coverage exceeded 150X; the UM bait design included additional sequencing specifically for *AR* and *FOXA1*, which resulted in enhanced coverage of these genes. We also assessed a panel of 134 cancer genes that, when altered, may be clinically actionable: defined as predictive for response or resistance to therapy, and/or with prognostic or diagnostic relevance [19]. Though read depth varied substantially, with some genes exhibiting deeper coverage in UM sequencing and others exhibiting deeper coverage in Broad sequencing, for all but two genes in the UM set (*NPM1*, *RHEB*) and five genes in Broad set (*NKX2-1*, *STK11*, *MAP2K2*, *CEBPA*, *ARAF*), read-depths exceeded 50X (Figure 2E).

### Tumor exome analysis: Mutations

The tumors evaluated in this study varied substantially in the number of non-synonymous somatic mutations ranging from 852^B^/1203^UM^ in SC_9008, which harbors an *MSH2* mutation that likely underlies this hypermutation phenotype [20], to 42^B^/57^UM^ in SC_9009 (Supplementary Tables 1, 2). Since mean target coverage was different at the two sites, which impacts power to detect mutations at lower allelic fractions and thus can confound comparison analyses, mutation comparisons were performed with a focus on adequately powered genetic loci [19, 21]. Of the mutations originally called in the SC_9008 tumor in the UM analysis, 88.5% of adequately powered events were concordantly called in the Broad-sequenced tumor, whereas 11.5% were powered to detect a mutation but the mutation was not identified (Figure 2F). In this tumor, 93% of the mutations originally called in the Broad analysis were adequately powered and validated in the UM analysis with 7% of the mutations adequately powered but not identified (Figure 2G). Analyses of the mutations in the other two tumors yielded similar rates of reproducibility. Collectively, these results may reflect differences in capture reagents, depth of coverage, analytical methods used for variant identification, or true biology in terms of intra-tumor heterogeneity.

Examination of variant calls from each center was performed for variants with at least 30X depth and an allelic fraction of 0.1 or higher, acknowledging that a majority of alterations present in one set but not the other is a result of insufficient power to call the variant. In both cases, the majority of nonsynonymous alterations identified in one set but not the other (374/613 [61%] for UM and 206/294 [70%] for Broad) were classified as short insertion/deletion events, consistent with prior reports that note challenges in reproducibly identifying this type of variant [22].

Several mutations were detected in genes with functional roles in prostate or other cancers. The exome of SC_9008 included an *AR* mutation, T878A that broadens ligand specificity, an inactivating *TP53* mutation, and a recurrent mutation in *SPOP*. A frameshifting indel disrupting *MSH2* was identified which likely contributed to the hyper-mutated genome of this tumor. The exome of SC_9010 had a point mutation in *ZFHX3/ATBF1*, a tumor suppressor gene previously reported to be recurrently inactivated in prostate cancers [23–25, 16]. Each of these pathogenic mutations was identified in both the UM and Broad analyses. A *PTEN* frameshift mutation (p.L296fs; allelic fraction 0.8) that accompanied a *PTEN* copy loss in SC_9008 was only identified in the initial UM insertion/deletion analysis, but was confirmed following manual review in both sequence data sets.

### Tumor exome analysis: Genome structural alterations

**Figure 3:**
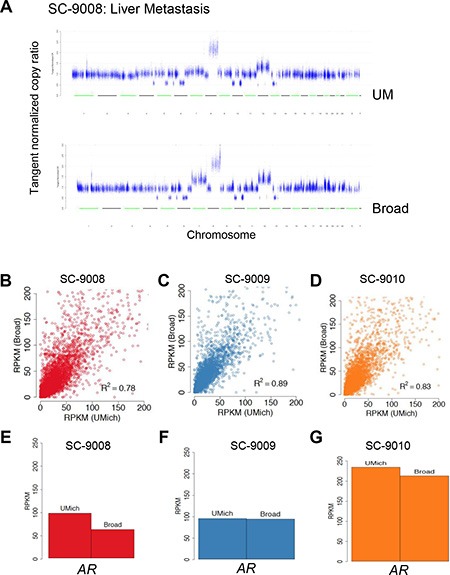
Comparison of DNA copy number assessments and RNAseq between sequencing centers A representative copy number profile obtained from UM and Broad from one case is show in (**A**). Reads per kilobase per million (RPKM) values from transcriptome data derived at each sequencing center for the three tumors are shown in (**B–D**) RPKM values for AR from each of the tumors is shown in (**E–G**).

To identify regions of the genome with allelic copy loss or gain, we assessed the exome sequence data using segmentation derived from copy ratios (See Supplementary Methods). Overall, there were substantial regions of copy gain and loss in each of the tumors. Overlays of the chromosome plots showed nearly identical patterns across the tumor genomes (Figure 3A; Supplementary Figure 3). Notable alterations in SC_9008 included a single copy loss of *APC*, *PTEN* loss, *RB1* loss, and focal amplifications of 8q that included the *MYC* locus. In SC_9010 notable alterations included *JAK2* loss on Chr9 and a copy gain of the *AR*. Each of these alterations was called in the UM and Broad analyses.

### Tumor RNAseq

RNAseq was performed to assess gene expression and identify gene rearrangements that encode fusion transcripts. Globally, gene expression concordance was significant between the transcriptomes from each tumor sample (r^2^ ≥ 0.78; Pearson) (Figure 3B–3D). Each tumor was found to exhibit high levels of transcripts encoding the AR (Figure 3E–3G) and AR-regulated genes such as *KLK3*/PSA and *TMPRSS2*, indicating an active AR transcriptional program, an important clinical finding for prioritizing therapeutic options.

Paired-end sequencing reads were used to identify fusion transcripts. Tumor SC_9009 expressed a fusion transcript involving the 5′ exons of *TMPRSS2* and 3′ exons of *ERG*, consistent with the commonly observed *TMPRSS2*-ETS family rearrangements that occur in 40– 60% of prostate cancers [26]. Both sequencing centers identified this rearrangement. To further investigate the concordance of fusion detection within and between samples, secondary multi-caller analysis was performed on transcriptome data from each tumor sample (see Methods). There was minimal overlap of fusion transcripts between two fusion callers applied to the same sample (See Supplementary Figure 4). Among putative fusions identified by both algorithms within a given sample from Broad (*n* = 12), the cross validation rate for the corresponding UM sample was 75% (9/12); among fusions identified by both callers in the UM samples (*n* = 18), the cross-validation rate for the corresponding Broad sample was 50% (9/18). However, in each tumor, numerous fusions were detected, the vast majority of which were unique to each tumor, have not been previously reported, and are of unclear significance.

### Exome assessments of germ line variants

To facilitate the accurate determination of somatic mutations and copy number alterations in a tumor sample, sequencing of germline DNA is often performed in a parallel assay. Incidental but clinically-useful findings unrelated to the intended assessment of cancer-associated alterations may be identified. To address how these incidental or secondary findings are disseminated, the American College of Medical Genetics and Genomics (ACMG) has established a list of 56 genes associated with 24 inherited conditions that should be reported [10]. Of these genes, 21 have clear causal roles in the inherited predisposition to neoplastic disease including *BRCA2* and the Lynch Syndrome DNA mismatch repair genes *MLH1*, *MSH2*, and *MSH6* [27–29].

We assessed each of the 56 ACMG genes in the whole exome data obtained from the corresponding benign tissue, and compared the read depth and sequence calls between the two sequencing centers (Table 1 and Supplementary Figure 2). Of the 56 genes, both the UM and Broad germline exomes provided > 50X coverage for all 56 ACMG genes, with only one exception (SDHC in UM exomes) (Supplementary Figure 2D). Since germline variant detection power does not require as substantial depth as tumor sequencing, this would not impact germline-focused clinical variant detection. One heterozygous variant, p.E1317Q, in the *APC* gene associated with a very modest 1.4-fold increased risk of colorectal cancer [30] was identified in the germline of SC_9010 by both sequencing centers (Supplementary Table 3).

## DISCUSSION

Comprehensive genomic assessments are increasingly used in clinical oncology in order to provide an appraisal of molecular alterations that have the potential to influence therapeutic decisions involving the selection of treatment [15]. Though the concept of genomic sequencing is understood at a fundamental level by providers and well-informed patients, there are numerous variations in the actual methodology that can influence reportable results. These include depth of sequencing coverage, the type of capture reagents used for exome and RNA analyses, whether target-based approaches are employed, disparities in tumor purity, and the integrity of DNA and RNA.

Our objective was to assess the consistency of ascertaining genomic information from tumors and corresponding germline DNA across different sequencing centers. We did not pre-specify the type of sequencing technology, the depth of sequencing, or any other parameter. Each of the two centers followed their standard operating procedures without an attempt to follow a common protocol.

Overall, the concordance in identifying key putative oncogenic aberrations was extremely high with only a *PTEN* frameshift mutation and a *MSH2* inactivating gene rearrangement identified by one center that accompanied a *PTEN* copy loss and *MSH2* inactivating indel, respectively, identified by both centers (Table 2 and Supplementary Table 4, 5). The consistency of reporting non-synonymous mutations ranged from 88.5% in a hypermutated tumor to 96.1% in a tumor with 54 non-synonymous mutations. Of the discordant mutations, the vast majority had sufficient depth of coverage to identify a mutation if present, and thus likely represents intratumoral heterogeneity. As two different portions of each particular metastasis were evaluated, rather than precisely the same tumor fragment, some degree of heterogeneity was expected. However, a focused analysis of 13 genes recurrently mutated in prostate cancer and 150 others with known oncogenic roles across human cancers determined 100% concordant mutation calls, indicating that the practical implications of intratumoral heterogeneity in terms of driver mutations and actionable variants, may be limited, at least in the context of metastatic disease [18].

**Table 2:** Comparative summary of cancer-associated findings from tumor SC_9008

EVENT	UM	BROAD
**Gene Copy Number**		
APC	Copy Loss	Copy Loss
AR	Amplification	Amplification
8q	Copy Gain	Copy Gain
PTEN	Copy Loss	Copy Loss
RB1	Copy Loss	Copy Loss
**Mutation**		
Mutations	1203 NSVs	852 NSVs
AR	p.T878A	p.T878A
TP53	p.R273C	p.R273C
SPOP	p.F102C	p.F102C
NCOR2	p.E1431K;indel	p.E1431K
ASXL2	p.R591C	p.R591C
PBRM1	p.Y1009H	p.Y962H
ARID1B	p.R1885H	p.R1885H
ARID2	p.A1773V	p.A1773V
MSH2	indel	indel
APC	indel	indel
NCOR1	indel	N.D.
**Expression**		
AR	High	High
KLK2	High	High
MSH2	MSH2-FSHR-fusion	N.D.
**Germline**		
56 AGMC Genes	No Pathological Variants	No Pathological Variants

N.D.; not detected; NSV; non-synonymous nucleotide variant.

Transcript levels measured by RNAseq were highly concordant across the sequencing centers. All tumors demonstrated high levels of AR-regulated transcripts including KLK3/PSA, TMPRSS2 and NKX3.1 in addition to the AR itself. RNAseq analyses in both centers identified fusion transcripts including a common rearrangement between TMPRSS2 and ERG in one tumor, though the detection of other fusion transcripts varied substantially depending on the algorithm used to identify such transcripts. Of interest, an *MSH2* mutation was identified in one tumor by both sequencing centers and likely contributed to the hypermutated genome. One sequencing center also identified a rearrangement involving *MSH2* predicted to inactivate the second *MSH2* allele. While alterations in *MSH2* occur as a heritable influence on cancer development in Lynch Syndrome [31], a germline *MSH2* mutation was not identified in the exome analysis from this patient.

In contrast to assessments of somatic genomic events in tumors where variation in tumor purity and tumor heterogeneity have the potential for influencing sequencing results, the analyses of germline DNA should consistently identify genomic variants. Of the 56 genes with pathogenicity as determined by the ACMG, all exons were covered to 30X for single nucleotide variant discovery. One likely pathogenic mutation in the *APC* gene was identified. This result is consistent with the anticipated rate of reportable incidental findings approximating 1–3% for this cohort of genes [10, 32].

Collectively, the findings from this study demonstrate that the results of somatic and germline sequencing are highly concordant across sequencing centers that have substantial experience in the technological requirements for preparing, sequencing and annotating DNA and RNA from human biospecimens. An aspect of genomic sequencing distinct from other assays used in clinical medicine is the breadth of data produced that encompasses anticipated drivers of disease as well as important incidental findings that may have health implications beyond the intended use of the original test. Further, a distinctive feature of oncology involves the iterative sequencing of tumor DNA and RNA, either directly from biopsies or potentially from circulating tumor cells or cell-free DNA, repeatedly over time to evaluate mechanisms of treatment resistance and the emergence of new targets. Establishing the reproducibility of genome-based assays is an essential step in routine use of this technology in research and clinical care.

## MATERIALS AND METHODS

### Tissue acquisition and preparation

Metastatic tumor samples were obtained from patients with castration resistant prostate cancer following written consent [14]. All samples were reviewed by pathologists with expertise in interpreting prostate cancer histology (Xiaotun Zhang and Lawrence True). Frozen tumor pieces containing > 70% tumor cells were processed as follows: for each frozen tumor block, a frozen section was cut, stained with hematoxylin and eosin and the percentage of tumor cells was ascertained by microscopy. The tumor block was then trisected and a second frozen section was taken from the bottom of each specimen, stained with hematoxylin and eosin and tumor cell percentage was confirmed. One tumor sample (approximately, 1/3 of the original specimen) was then sent to the Broad Institute, one tumor block (1/3 of the original specimen) was sent to the University of Michigan, and the remainder was retained at the University of Washington.

### Library preparations and sequencing

### Whole exome – Broad Institute

The preparation of libraries for massively parallel sequencing was performed as previously described [15, 33]. Detailed methods are provided in eMethods online. Each pool of whole exome libraries was subjected to paired 76 bp runs on a HiSeq 2000 sequencer. A BAM file was produced with the Picard pipeline (http://picard.sourceforge.net/), which aligned sequences to the hg19 human genome build.

### Whole exome – university of michigan

Tumor genomic DNA and total RNA were purified from the same sample using the AllPrep DNA/RNA/miRNA kit (QIAGEN). Libraries were sequenced with 100 bp paired reads on an Illumina HiSeq 2500 and aligned to the hg19 human genome reference.

### Transcriptome – broad institute

RNA was extracted from frozen tissue using the miRNeasy Mini kit (Qiagen). An automated variant of the Illumina Tru Seq™ RNA Sample Preparation protocol (Revision A, 2010) was used. Flowcell cluster amplification and sequencing were performed according to the manufacturer's protocols using either the HiSeq 2000 v3 or HiSeq 2500. Each run was a 76 bp paired-end with an eight-base index barcode read.

### Transcriptome – university of michigan

Transcriptome libraries were prepared using Agilent SureSelect Human All Exon V4 reagents and protocols. Libraries were sequenced using 100 bp paired-end reads on an Illumina HiSeq 2500.

### Nucleotide variant detection

### Broad institute

MuTect [21] was used to identify somatic single-nucleotide variants. Indelocator (http://www.broadinstitute.org/cancer/cga/indelocator) was applied to identify small insertions or deletions. Annotation of identified variants was done using Oncotator (http://www.broadinstitute.org/cancer/cga/oncotator).

### University of michigan

Paired-end reads were aligned using Novoalign v 3.02.00 and sorted using Novosort (Novocraft Technologies). Variants in both normal and tumor libraries were identified using the local realignment haplotype-based caller FreeBayes [34].

### RNA/transcript abundance

### Broad Institute

Gene expression was quantified using RNASeqQC [35].

### University of Michigan

Gene expression, as fragments per kilobase of exon per million fragments mapped was calculated using Cufflinks [36].

### Gene rearrangements/fusion transcripts

### Broad Institute

Fusion transcripts were originally identified using Prada [37]. Resulting putative fusion transcripts were manually reviewed.

### University of Michigan

Paired-end transcriptome sequencing reads were aligned to the human reference genome (GRCh37/hg19) using a RNA-Seq spliced read mapper Tophat2 [38] (Tophat 2.0.4). Fusion candidates were manually reviewed.

Multi-caller comparisons were subsequently performed using STAR Fusion and Tophat-Fusion [39].

### Copy number alterations

### Broad Institute

Copy ratios were calculated by dividing the tumor coverage by the median coverage obtained in a set of reference normal samples. The resulting copy ratios were segmented using the circular binary segmentation algorithm (36). Genes in copy ratio regions with segment means of greater than log_2_ (4) were evaluated for focal amplifications, and genes in regions with segment means of less than log_2_ (0.5) were evaluated for deletions.

### University of michigan

Copy number aberrations were quantified and reported for each gene as the segmented normalized log2-transformed exon coverage ratios between each tumor sample and matched normal sample [40]. The resulting copy ratios were segmented using the circular binary segmentation algorithm [41].

### Germline mutation calls

### Broad institute

Germline variants were identified using Unified-Genotyper [42].

### University of michigan

Germline variants were identified using FreeBayes [34].

## SUPPLEMENTARY MATERIALS FIGURES AND TABLES








