# Supplementary material for: A comparative assessment of clinical whole exome and transcriptome profiling across sequencing centers: implications for precision cancer medicine
Source: Oncotarget. 2016 May 5;7(33):52888–99. doi: 10.18632/oncotarget.9184 (PMC5288156; doi:10.18632/oncotarget.9184)
Supplement: Supplementary file 1 [file oncotarget-07-52888-s001.pdf]

A comparative assessment of clinical whole exome and transcriptome profiling across sequencing centers: Implications for precision cancer medicine

Supplementary Materials

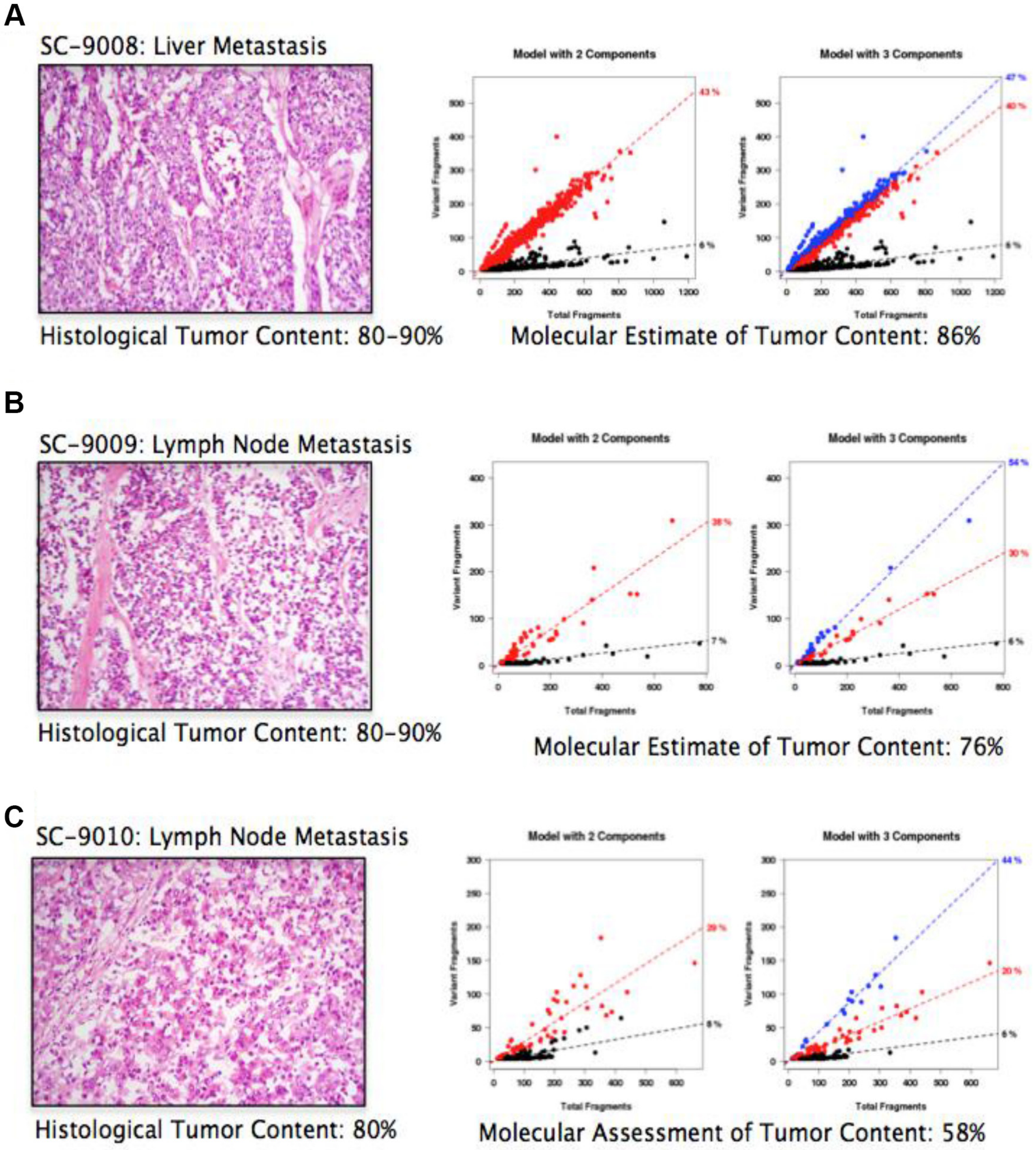

Supplementary Figure S1: Histological and sequence based estimates of tumor purity. Hematoxylin and eosin images of frozen sections taken from each of three metastatic prostate cancer samples, SC-9008, SC-9009, and SC-9010. Tumor estimates were made by a pathologist with expertise in prostate cancer histology and ranged from 80–90% tumor content. See eMethods for exome sequence-based approach of tumor content.

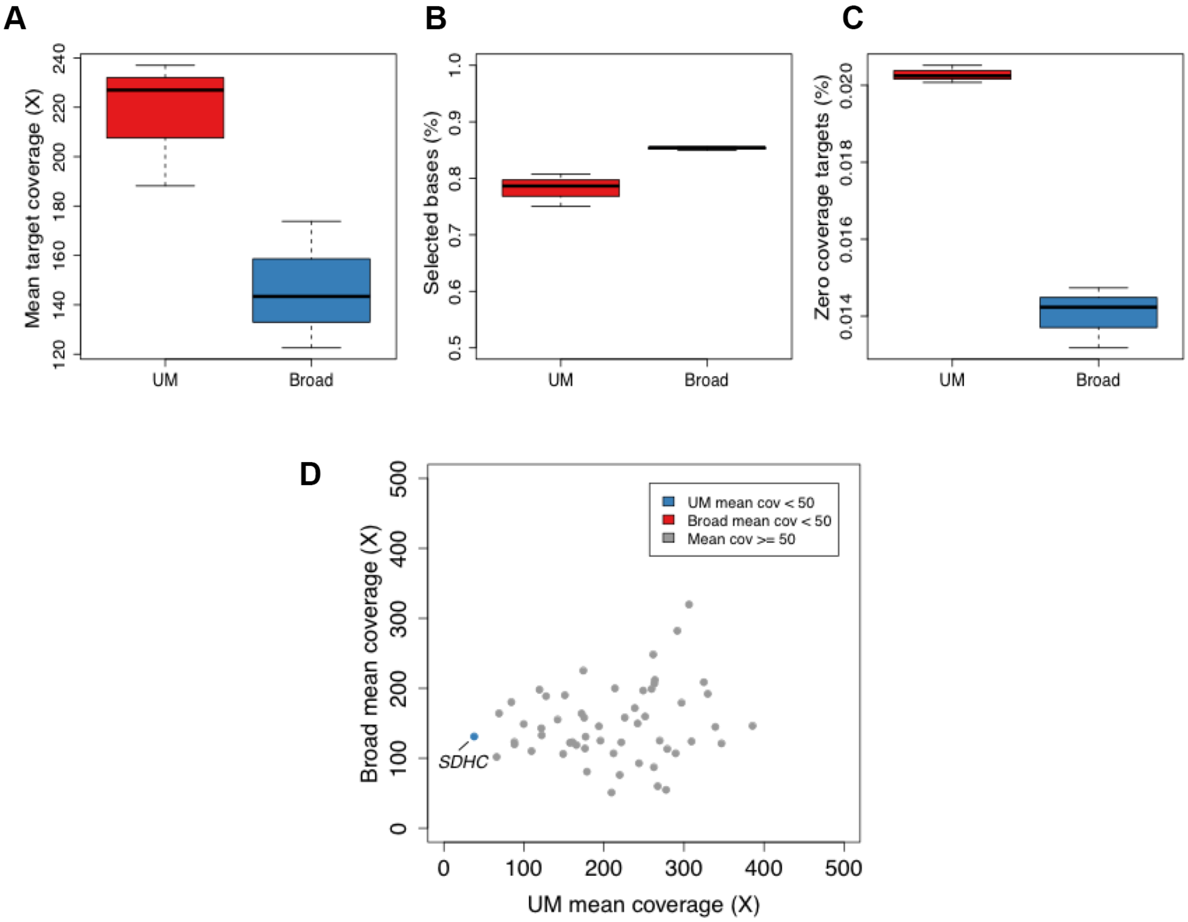

Supplementary Figure S2: Comparison of germ-line sequencing metrics across sequencing centers. Whole exome sequencing was performed on DNA extracted from benign tissues samples from each of three patients with metastatic prostate cancer. Shown are the sequencing data for mean target coverage (**A**); coverage for selected bases (**B**); and percentage of targets with zero coverage (**C**) across both centers. The mean coverage for 56 specific genes with heritable disease ramifications that are recommended by the ACMG to be reported (**D**).

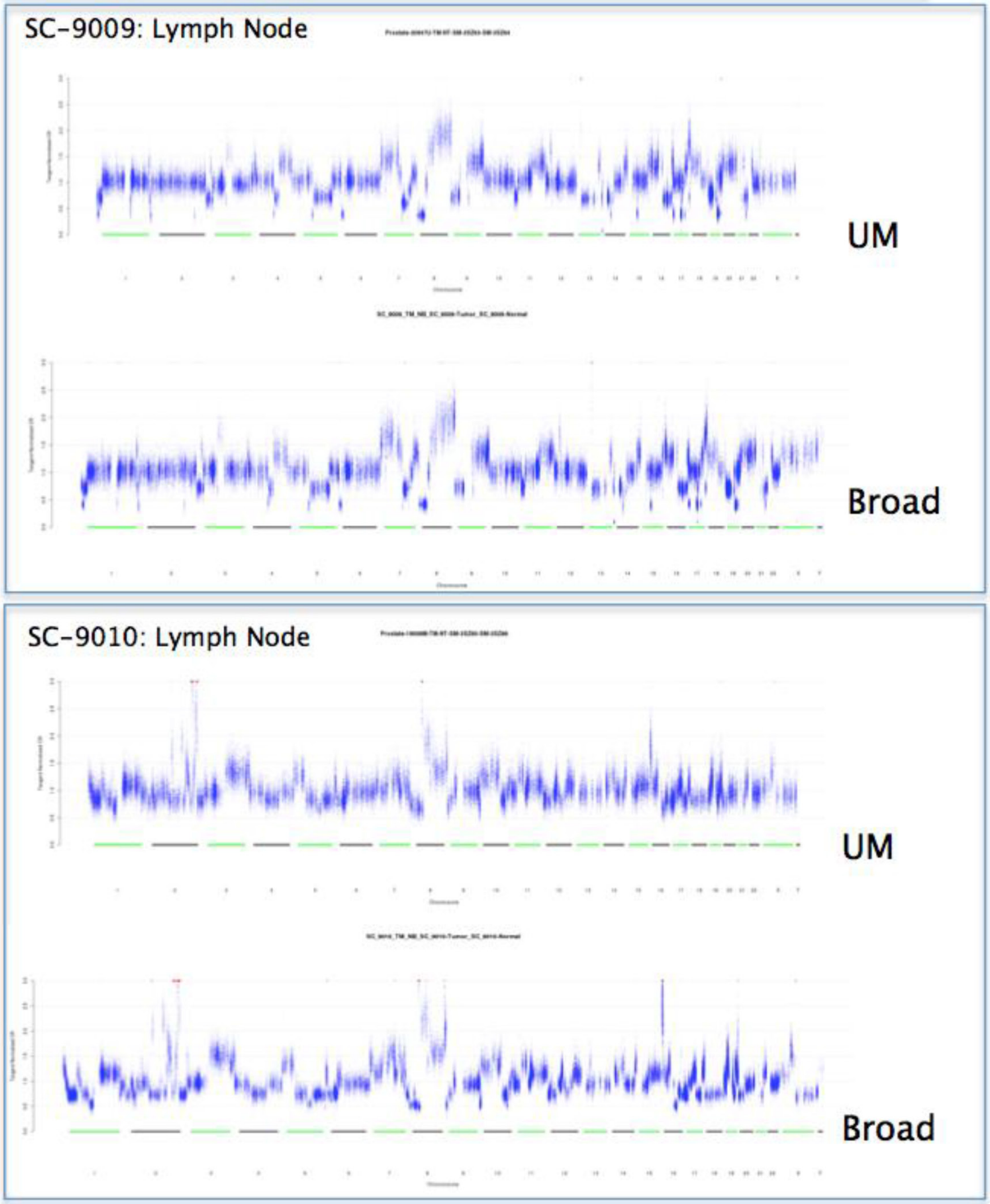

Supplementary Figure S3: Comparison of somatic DNA copy number losses and gains across the tumor genomes. Copy number variation plots were generated from whole-exome sequencing data from tumor SC-9009 and SC-9010 from the Broad Institute and University of Michigan (See Supplementary Methods).

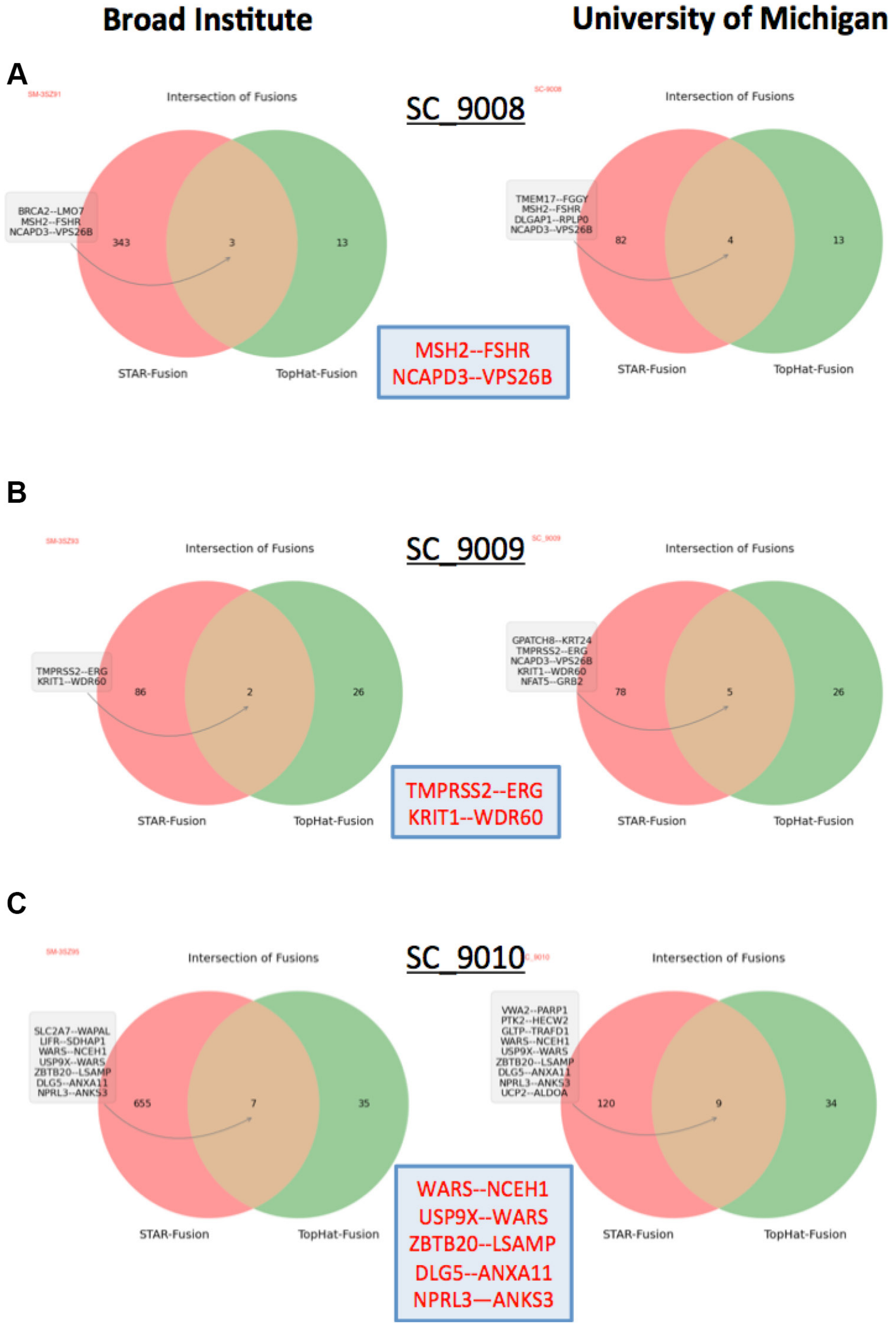

Supplementary Figure S4: Multi-caller fusion detection concordance. Two fusion detection algorithms (STAR Fusion and TopHat-Fusion) were applied to the same upstream raw transcriptome data generated by each site. For the three samples, the overlapping fusions within a given transcriptome and between transcriptomes derived from the same patient at the two sites are shown in (**A–C**).

Supplementary Table S1: Comparative summary of cancer-associated findings from tumor SC_9009

EVENT	UM	BROAD
**Gene Copy Number**		
General	Highly fragmented	Highly fragmented
**Mutation**		
Mutations	57 NSVs	42 NSVs
MLL2 (KMT2D)	p.S2373F	p.S2373F
IKBKB	p.T439I	p.T439I
**Expression**		
AR	High	High
KLK2	High	High
Fusion	TMPRSS2-ERG	TMPRSS2-ERG
Fusion	MTDH-RAD54B	N.D.
Fusion	ASCL2-CAMKMT	N.D.
Fusion	N.D.	KRIT1-WDR60
**Germ-Line**		
56 ACMG Genes	No Pathological Variants	No Pathological Variants

Supplementary Table S2: Comparative summary of cancer-associated findings from tumor SC_9010

EVENT	UM	BROAD
**Gene Copy Number**		
Chr2 Gain	STAT1;STAT4;ERBB4	STAT1;STAT4;ERBB4
Chr8 Gain	BAG4;FGFR1;LSM1;WH;SCL1L1	BAG4;FGFR1;LSM1;WH;SCL1L1
Chr9 Loss	JAK2	JAK2
ChrX Gain	AR	AR
**Mutation**		
Mutations	92 NSVs	47 NSVs
ZFHX3	p.E3441K	p.E3441K
**Expression**		
AR	High	High
KLK2	High	High
Fusions	Multiple	USP9X-WARS
**Germ-Line**		
56 ACMG Genes	APC: variant p.E1317Q	APC: variant p.E1317Q

Supplementary Table S3: Somatic mutations identified by WES: Broad

Supplementary Table S4: Somatic mutations identified by WES: UM

Supplementary Table S5: Germ-line variants of 56 ACMG disease-associated variants identified by whole exome sequencing

SUPPLEMENTARY METHODS

Tissue acquisition and preparation

Metastatic tumor samples were obtained from patients who died of castration resistant prostate cancer during a rapid autopsy performed within 6 hours of death. Patients and family members signed a written consent for the Prostate Cancer Donor Program at the University of Washington [1]. The Institutional Review Board of the University of Washington approved this study. Tumor fragments were embedded in OCT freezing medium. Frozen sections were examined to assess tumor purity following hematoxylin and eosin staining. Frozen tumor pieces containing > 70% tumor cells based on visual analysis were sent to the Broad Institute and University of Michigan for processing and sequence analyses.

Library preparations and sequencing

Whole exome-Broad Institute

DNA extraction was performed as previously described [2]. DNA libraries for massively parallel sequencing were generated as previously described. Libraries with concentrations above 40 ng/μl, as measured by a PicoGreen assay automated on an Agilent Bravo instrument, were considered acceptable for hybrid selection and sequencing. The exon capture procedure was performed as previously described. Libraries with concentrations between 40 and 50 ng/μL were normalized to 40 ng/μL, and 12.3 μL of library was combined with blocking agent, bait, and hybridization buffer. Finally, the hybridization reaction was reduced to 17 hours, with no changes to the downstream capture protocol. After post-capture enrichment, libraries were quantified using PicoGreen, normalized to equal concentration using a Perkin Elmer MiniJanus instrument, and pooled by equal volume on the Agilent Bravo platform. Library pools were then quantified using quantitative PCR (KAPA Biosystems) with probes specific to the ends of the adapters; this assay was automated using Agilent's Bravo liquid handling platform. Based on qPCR quantification, libraries were brought to 2 nM and denatured using 0.2 N NaOH on the Perkin-Elmer MiniJanus. After denaturation, libraries were diluted to 20 pM using hybridization buffer purchased from Illumina. Cluster amplification of denatured templates was performed according to the manufacturer's protocol (Illumina). HiSeq v3 cluster chemistry and flowcells, as well as Illumina's Multiplexing Sequencing Primer Kit. DNAs were added to flowcells and sequenced using the HiSeq 2000 v3 Sequencing-by-Synthesis method, then analyzed using RTA v.1.12.4.2 or later. Each pool of whole exome libraries was subjected to paired 76 bp runs. An 8-base index sequencing read was performed to read molecular indices, across the number of lanes needed to meet coverage for all libraries in the pool.

Sequence data processing: Exome sequence data processing was performed using established analytical pipelines at the Broad Institute. A BAM file was produced with the Picard pipeline (http://picard.sourceforge.net/), which aligns the tumor and normal sequences to the hg19 human genome build using Illumina sequencing reads. The BAM was uploaded into the Firehose pipeline (http://www.broadinstitute.org/cancer/cga/Firehose), which manages input and output files to be executed by GenePattern [3]. Quality control modules within Firehose were applied to all sequencing data for comparison of the origin for tumor and normal genotypes and to assess fingerprinting concordance. Cross-contamination of samples was estimated using ContEst [4].

Whole exome–University of Michigan

Matched normal genomic DNAs from frozen normal tissue blocks were isolated using the Qiagen DNeasy Blood & Tissue Kit, according to the manufacturer's instructions. Tumor genomic DNA and total RNA were purified from the same sample using the AllPrep DNA/RNA/miRNA kit (QIAGEN) with disruption using a 5 mm bead on a Tissuelyser II (Qiagen). RNA integrity was verified on an Agilent 2100 Bioanalyzer using RNA Nano reagents (Agilent Technologies). Whole exome capture libraries were constructed from 100 ng to 1 μg of DNA from tumor and normal tissue after sample shearing, end repair, and phosphorylation and ligation to barcoded sequencing adaptors (NEB). Ligated DNA was size selected for lengths between 200–350 bp and subjected to exonic hybrid capture using SureSelect Exome v4 baits (Agilent). Paired-end libraries were sequenced with the Illumina HiSeq 2500, (2 × 100 nucleotide read length. Reads passing the chastity filter of Illumina BaseCall software were used for subsequent analysis.

Transcriptome–Broad Institute

RNA was extracted from frozen tissue using the miRNeasy Mini kit (Qiagen) according to the manufacturer's instructions, including the optional on-column DNase digest. All samples were quantified using Nanodrop and quality was evaluated using Agilent's Bioanalyzer 2100. Total RNA was quantified using the Quant-iT™ RiboGreen® RNA Assay Kit (Invitrogen) and normalized to 4 ng/ul. An aliquot of 200 ng for each sample was transferred into library preparation which was an automated variant of the Illumina Tru Seq™ RNA Sample Preparation protocol (Revision A, 2010). This method uses oligo dT beads to select mRNA from the total RNA sample followed by heat fragmentation and cDNA synthesis from the RNA template. The resultant cDNA then goes through library preparation (end repair, base 'A' addition, adapter ligation, and enrichment) using Broad designed indexed adapters substituted in for multiplexing. After enrichment the libraries were quantified with qPCR using the KAPA Library Quantification Kit for Illumina Sequencing Platforms and then pooled equimolarly. The entire process is in 96-well format and all pipetting is done by either Agilent Bravo or PerkinElmer JANUS Mini liquid handlers.

Illumina Sequencing: Pooled libraries were normalized to 2 nM and denatured using 0.2 N NaOH prior to sequencing. Flowcell cluster amplification and sequencing were performed according to the manufacturer's protocols using either the HiSeq 2000 v3 or HiSeq 2500. Each run was a 76 bp paired-end with an eight-base index barcode read. Data was analyzed using the Broad Picard Pipeline which includes de-multiplexing and data aggregation

Transcriptome–University of Michigan

Transcriptome libraries were prepared using 200-1000 ng of total RNA isolated as above. Poly(A)+ RNA was isolated using Sera-Mag oligo(dT) beads (Thermo Scientific) and fragmented with the Ambion Fragmentation Reagent kit (Ambion, Austin, TX). cDNA synthesis, end-repair, A-base addition, and ligation of the Illumina indexed adapters were performed according to Illumina's TruSeq RNA protocol (Illumina). Libraries were size-selected for 250-300 bp cDNA fragments on a 3% Nusieve 3:1 (Lonza) agarose gel, recovered using QIAEX II gel extraction reagents (Qiagen), and PCR-amplified using Phusion DNA polymerase (New England Biolabs). The amplified libraries were purified using AMPure XP beads (Beckman Coulter). Total transcriptome libraries were prepared as above, omitting the poly A selection step and captured using Agilent SureSelect Human All Exon V4 reagents and protocols. Library quality was measured on an Agilent 2100 Bioanalyzer for product size and concentration. Paired-end libraries were sequenced with the Illumina HiSeq 2500, (2 × 100 nucleotide read length), with sequence coverage to 50 M paired reads and 100 M total reads. Reads passing the chastity filter of Illumina BaseCall software were used for subsequent analysis.

Analysis pipelines

Determination of tumor purity

Tumor content for each tumor exome library was estimated from the sequence data by fitting a binomial mixture model with two components to the set of most likely SNV candidates on 2-copy genomic regions. The set of candidates used for estimation consisted of coding variants that (i) exhibited at least 3 variant fragments in the tumor sample, (ii) exhibited zero variant fragments in the matched benign sample with at least 16 fragments of coverage, (iii) were not present in dbSNP, (iv) were within a targeted exon or within 100 base pairs of a targeted exon, (v) were not in homopolymer runs of four or more bases, and (vi) exhibited no evidence of amplification or deletion. In order to filter out regions of possible amplification or deletion, we used exon coverage ratios to infer copy number changes, as described below. Resulting SNV candidates were not used for estimation of tumor content if the segmented log-ratio exceeded 0.2 in absolute value. Candidates on the Y chromosome were also eliminated because they were unlikely to exist in 2-copy genomic regions. Using this set of candidates, we fit a binomial mixture model with two components using the R package flexmix, version 2.3-8. One component consisted of SNV candidates with very low variant fractions, presumably resulting from recurrent sequencing errors and other artifacts. The other component, consisting of the likely set of true SNVs, was informative of tumor content in the tumor sample. Specifically, under the assumption that most or all of the observed SNV candidates in this component are heterozygous SNVs, we expect the estimated binomial proportion of this component to represent one-half of the proportion of tumor cells in the sample. Thus, the estimated binomial proportion as obtained from the mixture model was doubled to obtain an estimate of tumor content.

Mutation calls (somatic)

Broad Institute

MuTect [5] was used to identify somatic single-nucleotide variants. Indelocator (http://www.broadinstitute.org/cancer/cga/indelocator) was applied to identify small insertions or deletions. Artifacts introduced by DNA oxidation during sequencing were computationally removed using a filter-based method [6]. Annotation of identified variants was done using Oncotator (http://www.broadinstitute.org/cancer/cga/oncotator).

University of Michigan

Paired-end reads were aligned using Novoalign v 3.02.00 and sorted using Novosort. (Novocraft Technologies) Variants in both normal and tumor libraries were identified using the local realignment haplotype-based caller FreeBayes [7].

RNA/Transcript abundance

Broad Institute

Gene expression was quantified using RNASeqQC [8].

University of Michigan

Gene expression, as fragments per kilobase of exon per million fragments mapped (FPKM; normalized measure of gene expression), was calculated using Cufflinks [9].

Gene rearrangements/fusion transcripts

Broad Institute

Fusion transcripts were identified using Prada [10]. Resulting putative fusion transcripts were manually reviewed.

University of Michigan

Paired-end transcriptome sequencing reads were aligned to the human reference genome (GRCh37/hg19) using a RNA-Seq spliced read mapper Tophat2 [11] (Tophat 2.0.4), with '—fusion-search' option turned on to detect potential gene fusion transcripts. In the initial

process, Tophat2 internally deploys an ultrafast short read alignment tool Bowtie (Version 0.12.8) to map the transcriptome data. Potential false positive fusion candidates were filtered out using ‘Tophat-Post-Fusion’ module. Further, the fusion candidates were manually examined for annotation and ligation artifacts. Junction reads supporting the fusion candidates were re-aligned using an alignment tool BLAT (http://genome.ucsc.edu/cgi-bin/hgBlat) to reconfirm the fusion breakpoint. Full length sequence of the fusion gene was constructed based on supporting junction reads, and evaluated for potential open reading frames (ORF) using an ORF finder (http://www.ncbi.nlm.nih.gov/gorf/gorf.html). Further, the gene fusions with robust ORFs and the amino acid sequences of the fused proteins were explored using the Simple Modular Architecture Research Tool (SMART) evaluating for gain or loss of known functional domains in the fusion proteins. Candidate fusion junctions with 4 or greater spanning reads were reported.

Copy number alterations

Broad Institute

Copy ratios were calculated for each captured target by dividing the tumor coverage by the median coverage obtained in a set of reference normal samples. The resulting copy ratios were segmented using the circular binary segmentation algorithm (Olshen 2004). Genes in copy ratio regions with segment means of greater than $\log_2$ (4) were evaluated for focal amplifications, and genes in regions with segment means of less than $\log_2$ (0.5) were evaluated for deletions.

University of Michigan

Copy number aberrations were quantified and reported for each gene as the segmented normalized log2-transformed exon coverage ratios between each tumor sample and matched normal sample [12]. To account for observed associations between coverage ratios and variation in GC content across the genome, lowess normalization was used to correct per-exon coverage ratios prior to segmentation analysis. Specifically, mean GC percentage was computed for each targeted region, and a lowess curve was fit to the scatterplot of log2-coverage ratios vs. mean GC content across the targeted exome using the lowess function in R (version 2.13.1) with smoothing parameter $f = 0.05$. The resulting copy ratios were segmented using the circular binary segmentation algorithm [13].

Germ-line mutation calls

Broad Institute

Germline variants were identified using UnifiedGenotyper [14].

University of Michigan

Germline variants were identified using FreeBayes [7].

REFERENCES

1. Morrissey C, Roudier MP, Dowell A, True LD, Ketchanji M, Welty C, Corey E, Lange PH, Higano CS, Vessella RL. Effects of androgen deprivation therapy and bisphosphonate treatment on bone in patients with metastatic castration-resistant prostate cancer: results from the University of Washington Rapid Autopsy Series. J Bone Miner Res. 2013; 28:333–340.
2. Fisher S, Barry A, Abreu J, Minie B, Nolan J, Delorey TM, Young G, Fennell TJ, Allen A, Ambrogio L, Berlin AM, Blumenstiel B, Cibulskis K, et al. A scalable, fully automated process for construction of sequence-ready human exome targeted capture libraries. Genome Biol. 2011; 12:R1.
3. Reich M, Liefeld T, Gould J, Lerner J, Tamayo P and Mesirov JP. GenePattern 2.0. Nat Genet. 2006; 38:500–501.
4. Cibulskis K, McKenna A, Fennell T, Banks E, DePristo M, Getz G. ContEst: estimating cross-contamination of human samples in next-generation sequencing data. Bioinformatics. 2011; 27:2601–2602.
5. Cibulskis K, Lawrence MS, Carter SL, Sivachenko A, Jaffe D, Sougnez C, Gabriel S, Meyerson M, Lander ES, Getz G. Sensitive detection of somatic point mutations in impure and heterogeneous cancer samples. Nat Biotechnol. 2013.
6. Costello M, Pugh TJ, Fennell TJ, Stewart C, Lichtenstein L, Meldrim JC, Fostel JL, Friedrich DC, Perrin D, Dionne D, Kim S, Gabriel SB, Lander ES, et al. Discovery and characterization of artifactual mutations in deep coverage targeted capture sequencing data due to oxidative DNA damage during sample preparation. Nucleic Acids Res. 2013; 41:e67.
7. Garrison E, Marth G. Haplotype-based variant detection from short-read sequencing. arVIX. 2012; 1207.3907.
8. DeLuca DS, Levin JZ, Sivachenko A, Fennell T, Nazaire MD, Williams C, Reich M, Winckler W, Getz G. RNA-SeQC: RNA-seq metrics for quality control and process optimization. Bioinformatics. 2012; 28:1530–1532.
9. Trapnell C, Roberts A, Goff L, Pertea G, Kim D, Kelley DR, Pimentel H, Salzberg SL, Rinn JL and Pachter L. Differential gene and transcript expression analysis of RNA-seq experiments with TopHat and Cufflinks. Nature protocols. 2012; 7:562–578.
10. Torres-Garcia W, Zheng S, Sivachenko A, Vegesna R, Wang Q, Yao R, Berger MF, Weinstein JN, Getz G, Verhaak RG. PRADA: pipeline for RNA sequencing data analysis. Bioinformatics. 2014; 30:2224-2226.
11. Kim D, Salzberg SL. TopHat-Fusion: an algorithm for discovery of novel fusion transcripts. Genome Biol. 2011; 12:R72.

12. Lonigro RJ, Grasso CS, Robinson DR, Jing X, Wu YM, Cao X, Quist MJ, Tomlins SA, Pienta KJ, Chinnaiyan AM. Detection of somatic copy number alterations in cancer using targeted exome capture sequencing. Neoplasia. 2011; 13:1019–1025.

13. Olshen AB, Venkatraman ES, Lucito R, Wigler M. Circular binary segmentation for the analysis of array-based DNA copy number data. Biostatistics. 2004; 5:557–572.

14. McKenna A, Hanna M, Banks E, Sivachenko A, Cibulskis K, Kernytsky A, Garimella K, Altshuler D, Gabriel S, Daly M, DePristo MA. The Genome Analysis Toolkit: a MapReduce framework for analyzing next-generation DNA sequencing data. Genome Res. 2010; 20:1297–1303.
